# Broadband CARS Hyperspectral Classification of Single Immune Cells

**DOI:** 10.1002/jbio.202400382

**Published:** 2025-01-18

**Authors:** Ryan Muddiman, Sarah Harkin, Marion Butler, Bryan Hennelly

**Affiliations:** ^1^ Department of Electronic Engineering Maynooth University Kildare Ireland; ^2^ Department of Biology Maynooth University Kildare Ireland; ^3^ Department of Computer Science Maynooth University Kildare Ireland

**Keywords:** BCARS, cell classification, coherent Raman

## Abstract

Broadband CARS is a coherent Raman scattering technique that provides access to the full biological vibrational spectrum within milliseconds, facilitating the recording of widefield hyperspectral Raman images. In this work, BCARS hyperspectral images of unstained cells from two different cell lines of immune lineage (T cell [Jurkat] and pDCs [CAL‐1]) were recorded and analyzed using multivariate statistical algorithms in order to determine the spectral differences between the cells. A classifier was trained which could distinguish the known cells with a 97% out‐of‐bag accuracy. The classifier was then applied to unlabeled samples containing a mixture of the two cell types on the same coverslip. This work demonstrates single‐cell analysis of pDCs (CAL‐1) and T cells (Jurkat) using BCARS. This approach enables an initial validation of cellular classification. We further demonstrate the capability of BCARS cell classification using single spectra of 5 ms acquisition time.

## Graphical abstract

We report on a novel approach to single immune cell classification using Broadband CARS hyperspectral imaging. This work implements a raster scanning approach and a custom semiautomated cell segmentation and preprocessing pipeline. Known cell types were used to train a classifier and then we use the trained model on a mixture of unlabeled cells on the same slide. We also provide methods to infer the classification performance on the unlabeled data. We also demonstrate that BCARS cell classification is possible using single spectra of 4000cm‐1 bandwidth with an acquistion time of 5ms.

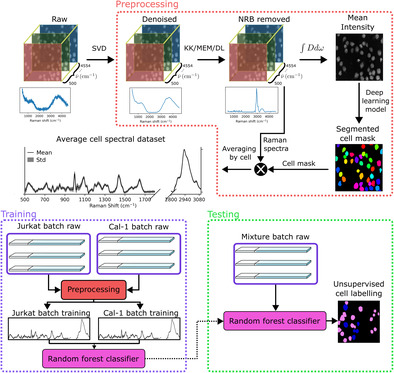

**Abbreviations**
ANA: antinuclear antibodies
APC: antigen‐presenting cells
IRF: interferon regulatory factor

## 1 Introduction

The application of label‐free spectroscopic methods toward biomedical applications is a promising area of research thanks to developments in new instrumentation methods such as coherent Raman spectroscopy (CRS) [1]. These methods can provide near instantaneous chemical contrast of single points of interest, with the potential to achieve live imaging rates [2]. Conventional spontaneous Raman (SR) spectroscopy utilizes a laser to interact with the vibrational bonds within molecules causing a shift in energy of the light. This energy shift results in a change of frequency in the scattered light and provides a molecular “fingerprint” spectrum that may be used to identify a substance. Coherent Raman spectroscopies are nonlinear optical techniques that similarly probe molecular vibrations; however, the main difference is that coherences between vibrational states are driven by the lasers and this results in a strong enhancement of the scattering signal. Stimulated Raman spectroscopy (SRS) in particular has received widespread attention due to its capacity for video‐rate imaging. However, recording broadband spectra with SRS requires complicated wavelength scanning approaches or multiple parallel lock‐in amplifiers. For this reason, Broadband Coherent anti‐Stokes Raman Spectroscopy (BCARS) has been demonstrated to be an attractive alternative for tissue imaging of biological matter with instantaneous spectral acquisition over wide bands from 400 to 4000 cm^−1^ [3, 4, 5, 6].

The BCARS electric field depends on the cube of the input electric field through the third‐order nonlinear susceptibility, $\chi^{\left(3\right)}$, resulting in a signal intensity on the order of approximately 104 times greater than SR [7]. This substantial increase in signal allows BCARS to achieve millisecond or even microsecond exposure times facilitating the recording of Raman hyperspectral images (HSIs) at the diffraction limit. Furthermore, BCARS is inherently free from fluorescence signals because the detected frequencies are higher than the incident light frequencies. Additionally, the technique achieves excellent depth selectivity; excitation is confined to a confocal volume due to the dependence on the cube of the input electric field. The major disadvantage of BCARS is the generation of an output field that arises from parametric mixing of the input fields via purely virtual states in the sample, and this results in a nonresonant background (NRB) $\chi_{\mathrm{NRB}}^{\left(3\right)}$, which coherently mixes with the resonant signal $\chi_{\mathrm{Res}}^{\left(3\right)}$. Such mixing can degrade the chemical information by obscuring the distinct resonant contributions. There have been several published methods to remove the NRB, allowing BCARS, in principle, to provide data comparable to that of SR with suitable processing. The most common methods include the Kramers–Kronig (KK) method and deep learning (DL), which are discussed in more detail later in the text.

It is well known that SR is capable of distinguishing between various cell pathologies [8, 9, 10]. Although developments in automating SR point‐measurements and applying statistical denoising [11, 12] have pushed SR toward 1 s acquisition times per cell for cytology, the throughput of these systems remains too slow for widespread application. A logical improvement in any Raman spectroscopy experiment is to increase the data acquisition rate as it allows for higher throughput analysis leading to many benefits. Thus, BCARS could potentially provide the acquisition rates that have previously limited the use of ST spectroscopy as a high‐throughput technique for cellomics.

BCARS spectroscopy of single cells has several limitations and this has hindered progress in obtaining the same success that SR has realized in this application area. Most notably, while BCARS is inherently confocal due to the nonlinear electric field dependence, the anti‐Stokes signal obtained depends on the ratio of the resonant to nonresonant susceptibility at that frequency [13]. In the fingerprint region, $\chi_{\mathrm{Res}}^{\left(3\right)}\ll \chi_{\mathrm{NRB}}^{\left(3\right)}$ due to low concentrations and small scattering cross‐sections, which results in a poor resonant signal strength. Theoretical estimates of the lower bound of concentration at which the CARS signal exceeds that from ST are estimated to be in the mM range [14]. This has steered research toward samples containing either a high localized density of scattering species or those with a high Raman cross‐section, either of which would increase the resonant susceptibility. Lipids are one example of a substance that satisfy both conditions and can thus be easily imaged using CARS [7, 15, 16, 17, 18].

BCARS studies on tissue imaging are more prevalent than single‐cell imaging, since the heterogeneous nature of biological tissue provides adequate chemical contrast among different structures, for example, collagen, myosin, nucleic acid, and so forth to which univariate approaches can be applied. As well as being more difficult to target because of their thin morphology compared with tissue, single cells have a much higher spectral similarity within and between cell type. This is because at the cellular level, there is a repeating of many of the same organelles and thus biomolecules in each cell. CARS hyperspectral imaging however has been shown to be capable of segmenting cellular organelles (nucleus, nucleolus, endoplasmic reticulum, and lipid droplets) based on the high‐wavenumber region alone [19]. Other notable studies using CARS to study single cells have primarily focused on the high‐wavenumber region as mentioned above [20, 21, 22].

In this article, we apply BCARS to obtain high‐speed chemical images of multiple cells (< 5 min per image of 20 × 200μ_m_) with the ultimate goal of automated high‐speed classification of slides. Chemical images not only provide molecular information at each point in the sample, but also morphological information, which can be used to increase the diagnostic accuracy of the analysis. Our approach used a pretrained cell segmentation algorithm to identify the area of the cell and to integrate all of the retrieved Raman spectra within the boundary of the cell in order to produce a single representative spectrum for each individual cell with higher SNR. This method provides a robust measurement of the cell fingerprint and prevents bias due to the sampling location which is inherent to point measurements. While this approach provides a rigorous validation of cell classification using BCARS, the effective acquisition time of the average cell spectrum is still in the order of a second and, therefore offers no obvious speed improvement over SR cell classification. However, using the single pixel spectra of 5 ms acquisition time we also achieve high sensitivity classification of the cell spectra. We believe that this paper includes several important contributions to the field of CRS and its application in biomedical research. These innovations include:

- We believe, we are the first to classify immune cells using high‐speed coherent Raman scattering making use of the fingerprint region of Raman spectra, which offers great potential for future cell and tissue classification studies.
- We have developed a method as an initial step in cell averaging to enhance the reliability of single‐cell classification previously developed with SR. This approach facilitates validation through morphological analysis of mixed samples.
- Building on this we achieve immune cell classification with a single spectral acquisition time of just 5 ms, which to the best of our knowledge sets a new benchmark for Raman‐fingerprint cell classification.
- This work paves the way for high‐speed BCARS flow cytometry.
- Based on recent advancements in stimulated Raman histology and virtual staining, our approach could also possibly allow for high‐speed classification of pathological slides based on biochemical features through broad grid scanning using BCARS.

## 2 Optical System and Physical Model

**FIGURE 1:**
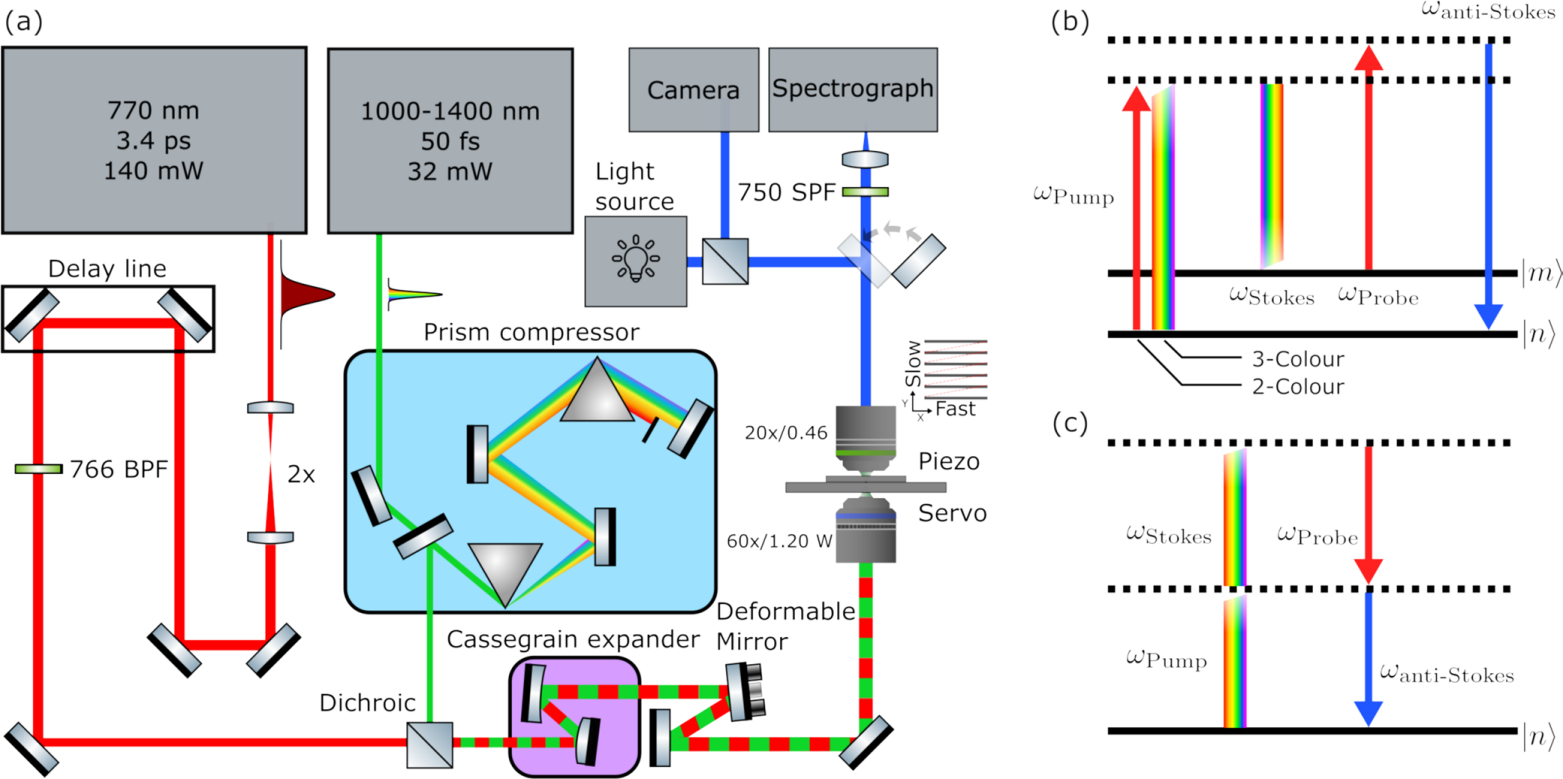
(a) Optical system diagram of the BCARS microscope, (b) Energy level diagram of the resonant four‐wave mixing process. Solid lines represent vibrational states of the molecule and dashed lines represent purely virtual states. (c) Energy‐level diagram of the nonresonant CARS process. In the energy‐level diagrams, the pump photon may originate from the SC laser, indicating the three‐color BCARS process, or the narrowband laser, indicating the two‐color BCARS process.

The BCARS optical system is illustrated in Figure 1 and incorporates a passively mode‐locked EDFA laser (Toptica) with a seed output at 1550 nm. The fundamental is frequency doubled using second harmonic generation in periodically poled LiNbO_3_ producing the narrowband probe beam at approximately 770 nm with a power of 140 mW. The seed is also coupled in to a highly nonlinear fiber for supercontinuum (SC) generation resulting in a broadband beam that spans 900–1400 nm at 32 mW. The probe beam is filtered using a band‐pass filter (FF01‐766/13‐25, Semrock) and expanded to approximately 2.4 mm in diameter. The SC beam is temporally compressed using an SF10 prism compressor and an automated phase retrieval routine [23]. Both the SC and probe are then combined using a dichroic (DMSP950, Thorlabs) and expanded using a Cassegrain telescope. The on‐sample probe and Stokes average power was 11 and 8 mW, respectively. Under these settings, there was no photodamage observed in any images. A motorized delay stage on the probe arm controls the relative delay between the probe and SC. The collinear beams are then sent to a deformable mirror (DMP40/M‐P01, Thorlabs) to correct for astigmatism introduced by the telescope. The beams are then coupled in to an upright transmission microscope (BX51, Olympus) utilizing a water immersion 1.2 NA/60× objective lens (UPlanSApo, Olympus) and 0.46 NA/20× collection lens (UmPlanFl, Olympus). The transmitted light is removed using a shortpass filter (FESH0750, Thorlabs), and the anti‐Stokes light is coupled in to a spectrograph (Shamrock 500i, Andor) using an achromatic doublet and detected using a thermoelectrically cooled CCD (Newton DU920P Bx‐DD, Andor) operating at −80°C. The spectrograph is equipped with a 300 L/mm grating resulting in a spectral resolution of approximately 10 cm^−1^. The sample stage consists of an XY servo stage (MS‐2000, ASI) for sample targeting and an XYZ piezo stage (P‐545.xR8S PInano, PI) for raster scanning. The spectral density of the SC results in the combined two/three color excitation and our measurement spans 500–4554 cm^−1^. The lack of any signal in the region corresponding to 2000–3000 cm^−1^ is due to the product of the frequency‐domain four‐wave mixing intensity at these frequencies being zero here for our laser system. All instruments were controlled using a custom script in MATLAB. More detail on the construction of this system is available in Ref. [24].

This BCARS system provides high‐speed vibrational imaging capabilities at the diffraction limit. In BCARS, the incident fields are termed the pump, Stokes and probe, at frequencies $\omega_{P}$, $\omega_{S}$, and $\omega_{\mathrm{pr}}$, respectively. The pump and Stokes cause a molecular coherence within the sample to oscillate at the difference frequency $\Delta =\omega_{P}-\omega_{S}$. The probe field may then scatter from this coherence, generating anti‐Stokes radiation at the frequency $\omega_{\mathrm{as}}=\omega_{P}-\omega_{S}+\omega_{\mathrm{pr}}$. If Δ is at a molecular vibrational frequency, the field interaction is enhanced. The resonant energy‐level diagram of the CARS process is shown in Figure 1b. Note that two physical models exist for the generation of the BCARS signal depending on which laser photon constitutes the pump. The first, known as the two‐color model, is the more traditional case for which the narrowband beam supplies the pump photon. This mechanism produces an enhancement of the Raman signal within the CH vibrational band. In the second case, known as the three‐color model, the SC supplies the pump photon and this mechanism leads to the generation of vibrational coherence primarily within the fingerprint spectral region. Notably, this configuration inherently results in no coherent enhancement within the spectral region void of Raman‐active modes for bio‐samples, often referred to as the silent region. In both the two‐ and three‐color models, the narrowband laser supplies the probe photon. More detail on the physical model for BCARS is available in Ref. [3]. When Δ is far from a resonance, the four‐wave mixing may still occur due to the finite value of the susceptibility from off‐resonant vibrations and electronic resonances and this results in the NRB. In this case, the fields only interact with virtual states of the molecule, effectively perturbing the electrons adiabatically. This process is shown in Figure 1c. The NRB can be considered both a blessing and a curse. While it is difficult to retrieve the Raman‐like spectrum from the mixed BCARS spectrum, the NRB can act as a local oscillator and the mixing may effectively amplify the Raman spectrum. Removal of the NRB is discussed in more detail in Section 4.4.

## 3 Sample Preparation and Recording Parameters

CAL‐1, a BPDCN (blastic plasmacytoid dendritic cell [pDC] neoplasm) cell line, was provided by Prof T. Maeda (Nagasaki University) [25, 26]. The Jurkat T‐cell leukemia cell line was provided by Dr. Martina Schroeder (Maynooth University). CAL‐1 pDC and Jurkat T cells were cultured in RPMI 1640 with GlutaMAX (Gibco, 61 870 010) supplemented with 10% FBS (Sigma, F7524). Cell lines tested negative for mycoplasma (Eurofins Genomics).

CAL‐1 pDC and Jurkat T cells were centrifuged at 1500 rpm for 5 min and resuspended in 1 mL Dulbecco's Phosphate Buffered Saline (PBS) (Sigma, D8537). Three samples were prepared containing a total of 5 × 10^5^ cells; CAL‐1 cells only, Jurkat T cells only, or 1:1 mix of both cell lines. Cells were centrifuged at 2300 rpm at 4°C for 5 min and supernatant was discarded. The cells were resuspended in 500 L of 10% formalin (0.2 μm filtered) and incubated for 10 min at room temperature. Fixed cells were washed in 1 mL of PBS, centrifuged as before and resuspended in 150 μL molecular grade water (Sigma, W4502). A volume of 50 μL was deposited on to a glass coverslip and allowed to dry at room temperature. In this work, we did not observe visual or spectroscopic degradation of using the dry mounting method. We note that we attempted suspended cell imaging and failed. This was due to optical trapping effects that caused movement of the suspended cells during scanning. This could possibly be circumvented using a cell adhesion aid such as poly‐L‐lysine, however, we did not use that approach, opting instead for the simpler drop‐dry deposition.

BCARS HSIs of each cell type were recorded (10 images each) using the system described in Section 2 at a pixel acquisition time of 5 ms using constant velocity raster scanning. During slow axis movement the full‐vertical binning (FVB) data were read from the detector. The temporal delay of the probe was set to approximately 0.5 ps relative to the SC. The deformable mirror astigmatism compensation was optimized using a measurement of the resonant CARS signal in the cytoplasm of a cell. The HSIs were of size 200 × 200 μm at 1 μm step size and with each pixel spanning the biological vibrational spectrum from 500 to 4554 cm^−1^. A dark current spectrum was also taken. The whole procedure was then performed on the mixture samples (12 images each).

## 4 Preprocessing for Hyperspectral Imaging

### 4.1 Wavenumber and Sensitivity Calibration

The spectrograph was wavelength calibrated using a neon lamp by the method presented in Ref. [27] and the probe laser wavelength was determined using a reference measurement from benzonitrile with a large probe delay. This resulted in practically no NRB since the coherence time of the NRB is on the order of fs while that of Raman transitions is on the order of ps [28, 29]. The wavenumber axis was then calibrated using the wavenumber conversion formula applied to the calibrated wavelength axis and the probe wavelength. Sensitivity calibration using a reference material or NIST‐calibrated white light is unnecessary because the KK algorithm makes use of a reference glass spectrum and in this process, the spectra are inherently corrected for variation in sensitivity caused by the spectrometer and optical system. Following wavenumber calibration, each HSI was then preprocessed to ensure data quality of the spectra as described in the following subsections.

### 4.2 Cosmic Ray Removal

Cosmic rays were detected using a thresholding of the mean spectral intensity of the raw HSI. Under the experimental conditions used, a cosmic ray event resulted in approximately less than one corrupted pixel per image, on average. The corrupted pixels consistently had a mean count > 10 times the average signal intensity. Therefore, this value was chosen as the detection threshold for each pixel. Any remaining cosmic rays would have been detected by the denoising procedure, for which none were observed. The de‐spiking was performed by mean replacement using the four nearest pixels. No edge pixels were corrupted. The replacement with an average was used since it is a single‐pixel method, other methods using principal component analysis (PCA) may introduce spectral bias across the dataset in pixels where no corruption has occurred.

### 4.3 Denoising

**FIGURE 2:**
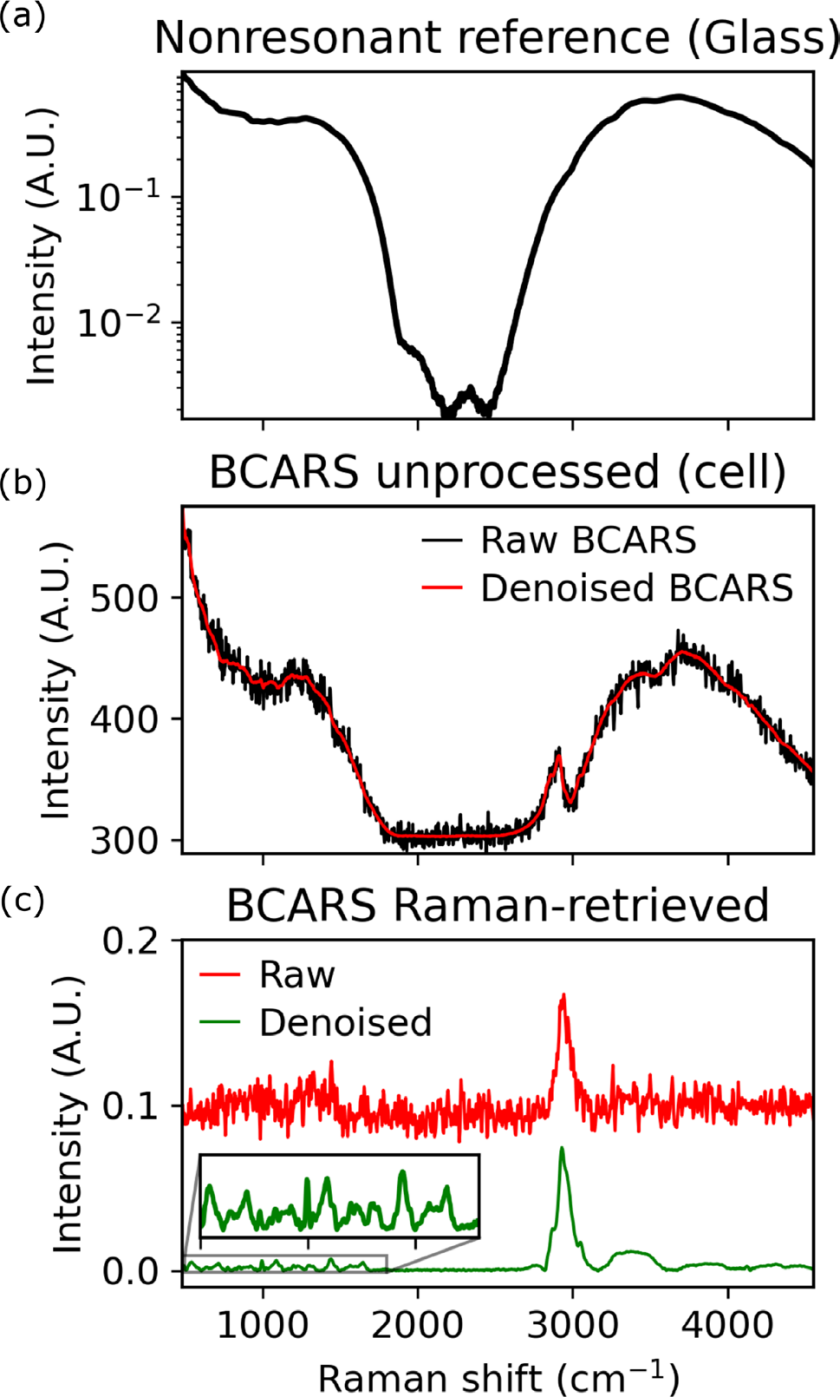
(a) Spectrum of the nonresonant background reference signal for a HSI, (b) Raw and denoised single cell BCARS spectrum prior to any further preprocessing, (c) Raman‐like spectrum obtained using KK using both the raw and SVD denoised signals shown in (b). The raw spectrum was scaled vertically for clarity.

Denoising was performed using the truncated singular value decomposition (SVD) with the specific singular values kept in the reconstruction based on a custom algorithm involving the 2D Fourier transform of the right singular vectors as described in the supplementary information of Ref. [30]. Denoising a BCARS HSI is a crucial step for effective retrieval of a Raman‐like signal since practical phase‐retrieval involves the ill‐conditioned application of operators such as the Discrete Hilbert‐Transform (DHT) in the KK method. In the presence of noise, the resulting retrieved signal can be completely obscured due to the instability of the process. Therefore, optimal denoising must be applied to obtain meaningful results. The truncated SVD is a popular method for denoising HSIs since it produces the closest low‐rank matrix to the original matrix, with the difference ideally being noise. Choice of the SVs to keep in the truncation is typically dictated by an empirical or theoretical threshold on each value of σ, below which, the singular values are set to zero. In this study, specific SVs were chosen if the corresponding singular vector contained spatial variation as determined by the 2D discrete Fourier transform of the right singular vectors. The effect of noise on a cell spectrum is shown in Figure 2. The reference NRB as shown in (Figure 2a) effectively describes the excitation profile of the laser system over which resonances will be measured. The raw and denoised signals from a cell are shown in (Figure 2b), while the resulting KK‐retrieved spectra are shown in (Figure 2c) for both an undenoised and denoised input spectrum. The effect of the noise on the output results in a completely corrupted signal.

### 4.4 NRB Removal

The most common methods of NRB removal incorporate a priori information about the form of the CARS spectrum, such as the KK relations, which relies on the response, $\chi^{\left(3\right)}$ being causal and therefore analytic [31]. Direct application of the KK relation however results in errors in the retrieved phase of $\chi^{\left(3\right)}$ due to several reasons. It has been shown that using baseline correction, the KK method can provide more reliable estimates of $\chi_{\mathrm{Res}}^{\left(3\right)}$ from ${\left|\chi^{\left(3\right)}\right|}^{2}$ [30]. Another type of NRB removal approach gaining in popularity is DL‐based approaches. DL utilizes many hidden layers in a network to learn features that enable the transformation between a corrupted spectrum to an NRB‐free spectrum. There have been several DL implementations applied to NRB removal in CARS, such as convolutional neural networks (CNN) [32, 33], Long short term memory (LSTM) networks [34], generative adversarial networks (GAN) [35] and convolutional autoencoders (CAE) [36, 37]. All NRB removal approaches aim to linearize the response and remove the spectral distortion of the peaks; however, DL has the added benefit of learning further information such as the spectral convolution from the lasers, noise from the detectors, and phase distortions, when adequate training data are available. Thus, DL approaches to NRB removal seem the obvious choice. However, the major drawback in DL is the training time, which compared with the numerical approaches can be orders of magnitude longer. In this study, the KK method was used since it is the most established technique in phase‐retrieval for BCARS.

All HSIs presented in this article were processed using the phase‐amplitude corrected KK method [30]. The average of the signal for the segmented background region for each image was used as the reference nonresonant background measurement. The smoothing parameter for the phase‐retrieval was set to 100 and the asymmetry parameter was set to 0.0001 for the high‐wavenumber region (2745–4554 cm^−1^), 0.001 for the fingerprint region (500–1708 cm^−1^), and 0.01 for the silent region (1708–2745 cm^−1^). The spectrum for each cell was then averaged resulting in a single cell spectrum. Residual inter‐sample variance due to scatter was removed from each spectrum using the EMSC algorithm with a fifth‐order polynomial and a mean reference BCARS spectrum [38]. Finally, each spectrum was cropped to a range of 500–3142 cm^−1^ and then normalized to its minimum and maximum value.

## 5 Methodology for BCARS Hyperspectral Single‐Cell Classification (HSCC)

**FIGURE 3:**
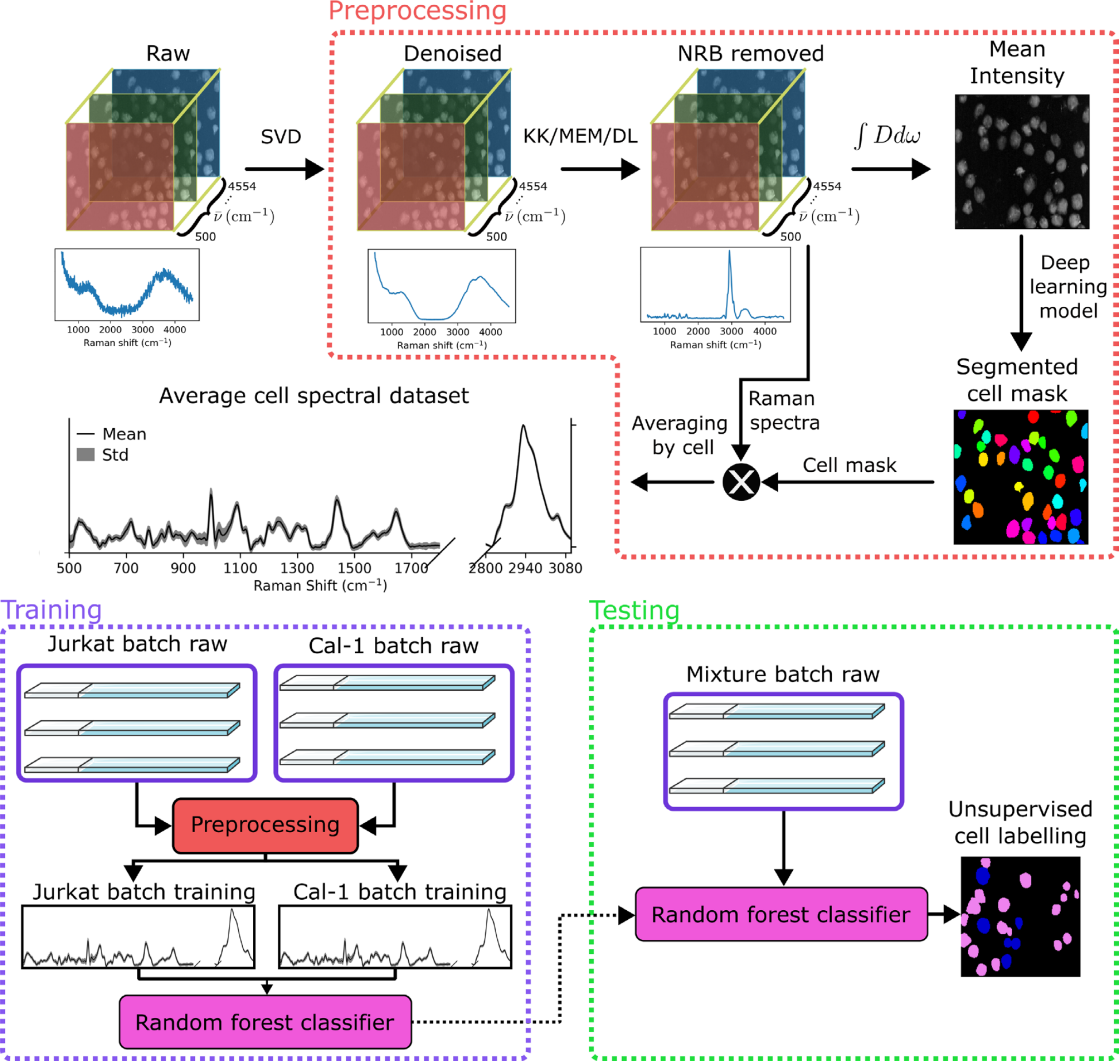
Flowchart of the experimental and data analysis pipeline. The various steps are described in the main text.

The workflow of BCARS‐HSCC is illustrated in Figure 3 and is summarized as follows: BCARS HSIs of two different isolated cell types, namely Jurkat T cells & CAL‐1 pDCs were obtained as described in the previous sections. The HSI contains information on the full spatial extent of the cell and therefore no targeting of cells was required, as is typically performed for Raman spectroscopy. In summary, the preprocessing step involves SVD denoising and KK‐based NRB removal. Preprocessing also includes a pretrained DL method that is applied to segment the cell regions using the total BCARS intensity image. The spectra within each of these segments were then integrated to produce a single spectrum representing each individual cell. In this way, known labeled datasets of single‐cell spectra were obtained from which a random forest (RF) classification model was trained in order to learn the features that distinguished the two cell types. Finally, HSIs of a mixed sample of the two cell lines at 1:1 concentration were also obtained in order to simulate the clinically relevant situation of multiple unknown cell types within a single specimen. The various steps are described in more detail below.

### 5.1 Step 1: Preprocessing

The BCARS‐HSCC preprocessing step is illustrated in Figure 3 and initially consists of first applying SVD denoising followed by KK‐NRB removal as described in more detail in the previous section. This is followed by integrating the resultant 3D dataset over the wavenumber dimension to obtain a 2D image of the cells. This total intensity image was then processed by the Cellpose 3 segmentation algorithm [39, 40] in order to segment each individual cell region. In order to obtain the NRB reference for KK, the raw denoised BCARS intensity image was integrated and the largest area mask was averaged to give the spectrum of the coverslip. Cellpose is a DL cell‐agnostic segmentation algorithm that does not require model retraining or parameter tuning. Classical approaches to segmentation such as the watershed algorithm often fail to segment cells that are in close proximity due to the basins overlapping. This was also the case for our experiment. The Cellpose model could accurately define cell masks even when the cells were visibly touching. The model used was “cyto‐3,” incorporating an image restoration step that produces the input to the segmentation algorithm with the aim of increasing the accuracy of the segmentation. Once the cell extents were obtained, the spectra for each cell were averaged using the mean spectrum inside the extents.

Noisy retrieved cell spectra within the labeled dataset were identified using Pearson correlation analysis between each cell sample and a high‐SNR sample obtained empirically. Samples with a Pearson correlation coefficient *ρ* < 0.995 were removed from the dataset. This resulted in 346 cells in total within the labeled set with a split of 1:1.7 (Jurkat T/CAL‐1). EMSC normalization was then performed and the resulting data normalized to its minimum and maximum per cell.

To perform an initial evaluation of the single‐cell spectra, PCA was first performed on the labeled dataset to visualize the spectral variance within the data. Using PCA, the variance between the known spectra of each cell type was visualized. We note that PCA was used only as a data evaluation method to visualize the differences between the spectral datasets and was not used as part of the multivariate classification model in Step 2.

### 5.2 Step 2: Training a Multivariate Classifier

A RF classifier was trained on the full labeled dataset using 40 decision trees and balanced class weighting to remove the bias from uneven frequency distributions of the training samples. The RF is an ensemble approach to supervised classification. Using bootstrapping of the input samples, a training set is built and split based on a series of binary decision trees. At each node, a sample is labeled using a randomly chosen feature. The model learns by maximizing the impurity decrease from each node to subsequent nodes. The same method was used in Ref. [11] to classify leukocytes. Hyperparameter tuning was performed to optimize the number of trees only, using 10‐fold cross‐validation. The RF model was implemented using the SciKit‐learn package in Python with default parameters except for those specified. The out‐of‐bag training score for the RF model was 97%. The feature importance of each spectral variable was calculated based on the mean Gini impurity decrease within each tree.

### 5.3 Step 3: Testing on Unlabeled Mixed Sample

The raw mixed HSIs were preprocessed using the method described in Step 1. The resultant dataset was input to the trained RF model in order to predict the class probability of each detected cell in the mixed dataset. The class probabilities were then used to classify each cell using a threshold of 0.5.

Separately as a form of validation, the predicted cell spectra were projected on to the PCs obtained from the labeled dataset in Step 1. Labels were applied based on the RF prediction. The resultant scatter plot of the PC scores was investigated for similarities to the corresponding result for the labeled dataset.

## 6 Results

### 6.1 Step 1: Labeled Cell Analysis

**FIGURE 4:**
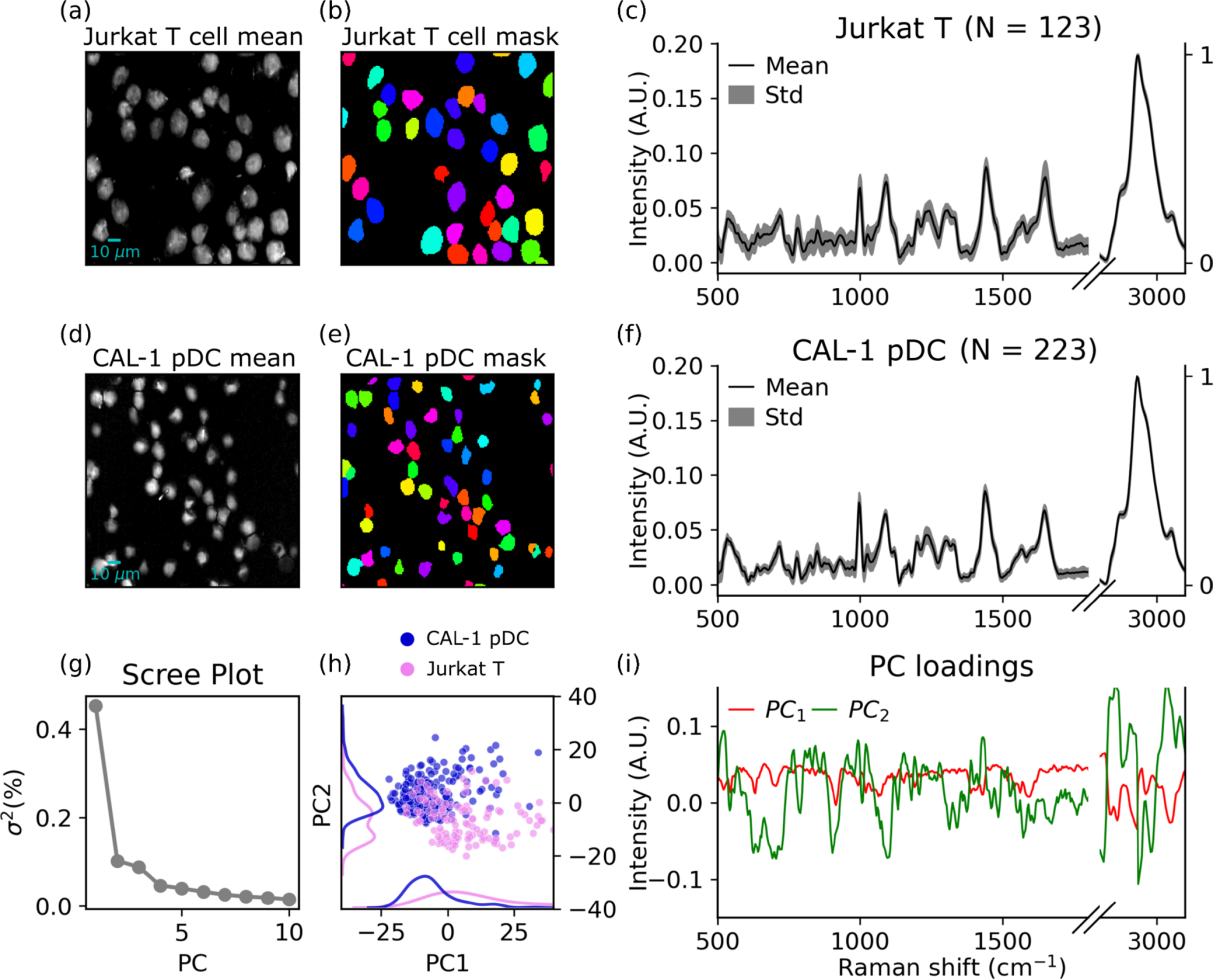
Supervised analysis of each cell type using PCA. (a) Mean intensity image from an image containing Jurkat T cells, (b) cell mask of image in (a), (c) mean and standard deviation of the Jurkat T‐cell dataset, (d) mean intensity image from an image containing CAL‐1 cells, (e) cell mask of image in (d), (f) mean and standard deviation of the CAL‐1 cells, (g) explained variance plot of cell data, (h) PCA score plot of cell data, (i) first two principal components (PCs) of cell data.

In Figure 4, the mean BCARS image and resulting cell segmentation mask is shown for two example HSIs of each labeled cell type. Each color in the mask corresponds to the extent of an individual cell. It can be seen from the segmented mask images that the segmentation was accurate even when cells were very close in extent. This usually leads to erroneous results in the watershed algorithm. There did not appear to be any misclassified cell extents within the whole dataset. The average retrieved spectrum for each cell type was also consistent with a SR spectrum from an immune cell. The relative intensity between the high‐wavenumber region and fingerprint region was similar to the expected ratio due to the relative abundance and cross‐sections of the modes in each region. The signal‐to‐noise ratio was visibly also much higher for the high‐wavenumber region, as expected, due to the larger average signal intensity in this region and lower relative NRB strength. The PCA scree plot indicating the percentage of the variance explained for each PC is also given. It was found that the first two PCs contributed to 55.4% of the variance within the data. The score of each cell in the PC space is also shown, with moderate overlapping of the two cell types. The first two PCs are plotted in order to show the variance of each dimension in the projection.

The supervised analysis of the labeled data showed discernible features in the Raman spectra typically attributed to known vibrational modes in eukaryotic cells. As shown in Figure 4, the two cell types are moderately separated on the first two PCs. However, there were no discrete clusters observed on the first two PCs. Inspection of the first PC shows that most variation was due to the high wavenumber region at 2934 and 3054 cm^−1^. On PC2 several peaks within the fingerprint region contributed to variation in the data, including the CH_2_ scissoring mode at 1440 cm^−1^, phenylalanine ring breathing mode at 1003 cm^−1^, adenine and guanine markers at 1575 cm^−1^, modes due to Uracil, Cytosine, Thymine ring breathing; O‐P‐O symmetric stretch at 784 cm^−1^ and the PO_2_ stretching mode at 1095 cm^−1^.

### 6.2 Step 2: Supervised Classifier Performance

**FIGURE 5:**
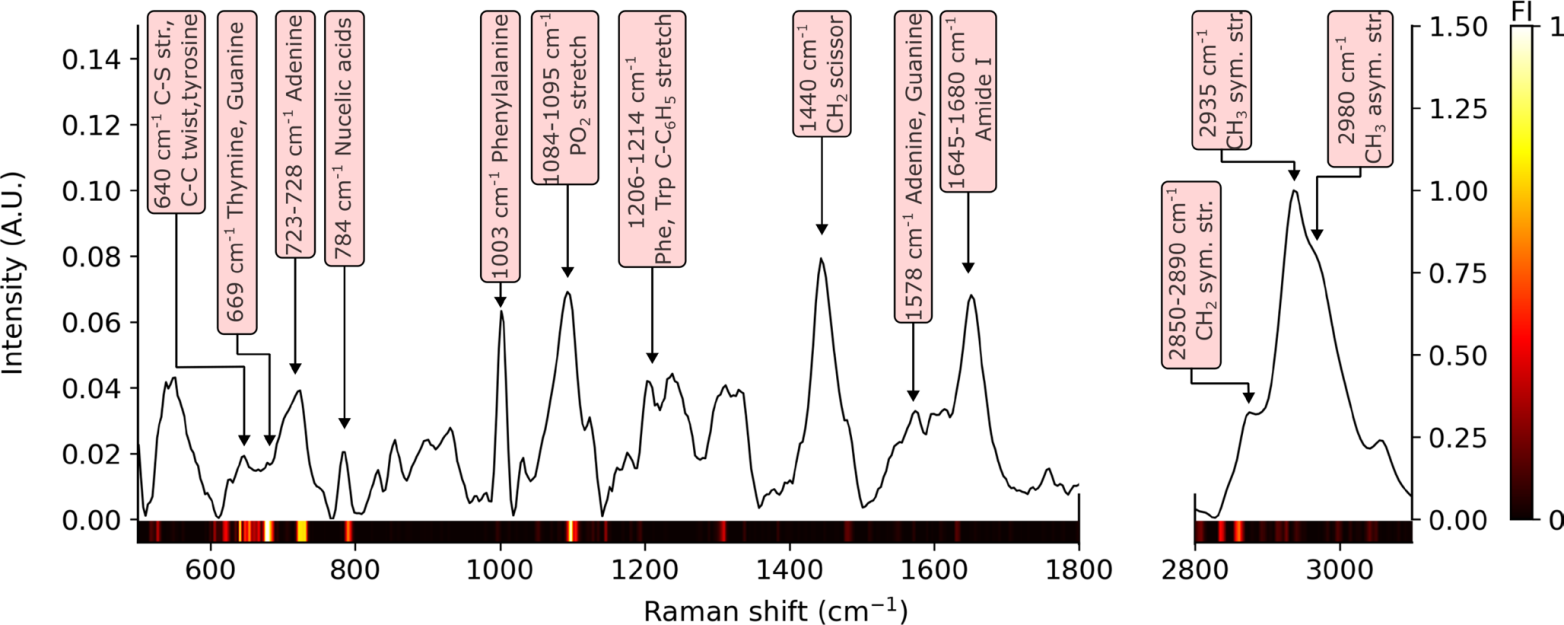
Single cell retrieved BCARS spectrum from a CAL‐1 sample. Vibrational peaks were assigned based on Ref. [41, 42]. Also shown is the normalized feature importance of the RF classifier.

An example retrieved broadband CARS spectrum from a CAL‐1 cell is shown in Figure 5. The spectrum has a very similar appearance to a SR spectrum from an immune cell with distinct peaks and features in the fingerprint region (nucleic acids, lipids, protein) and high wavenumber regions (symmetric and asymmetric stretching of CH_2_ and CH_3_). Peak assignment of the major bands in the cell measurement is shown in the figure. Since the high‐wavenumber region in the vibrational spectrum of a cell consists only of a small number of features (mainly alkyl groups) that broadly overlap, the diagnostic information is likely to be lower than for the fingerprint region. Analysis of the feature importance (FI) of the supervised RF classifier gives insight in to the specific wavenumbers that contribute to the classification. The method used here was a built‐in method calculated as the normalized decrease in impurity (measured as the randomness) of a node when a specific feature is used. The FI is a nonnegative number and higher values indicate a larger importance. Given that the full BCARS spectrum was used to train the RF model, inspection of the FI after training is a useful approach to feature analysis. The spectral FI of the trained model is shown in Figure 5. Corresponding high FI values that occur where vibrational resonances exist gives evidence toward their likelihood in contributing to the classification. It was observed that the highest FI value was in the fingerprint region, corresponding to the 669 cm^−1^ band which relates to the presence of nucleobases thymine and guanine. The next most prominent feature was the band occurring at 723–728 cm^−1^, relating to adenine. The rest of the spectral features are shown in Figure 5. There were also high FI values within the high‐wavenumber region, not particular to one band but multiple wavenumbers as seen in the figure. The supervised classification accuracy was measured using 10‐fold cross‐validation. The mean balanced accuracy obtained was 99.4% for the test set and 100% for the training set. The full data were used in the RF model and the out‐of‐bag error was 3%. The results of the cross‐validation are shown in Supporting Information S1

### 6.3 Step 3: Unlabeled Testing on Unlabeled Mixed Sample

**FIGURE 6:**
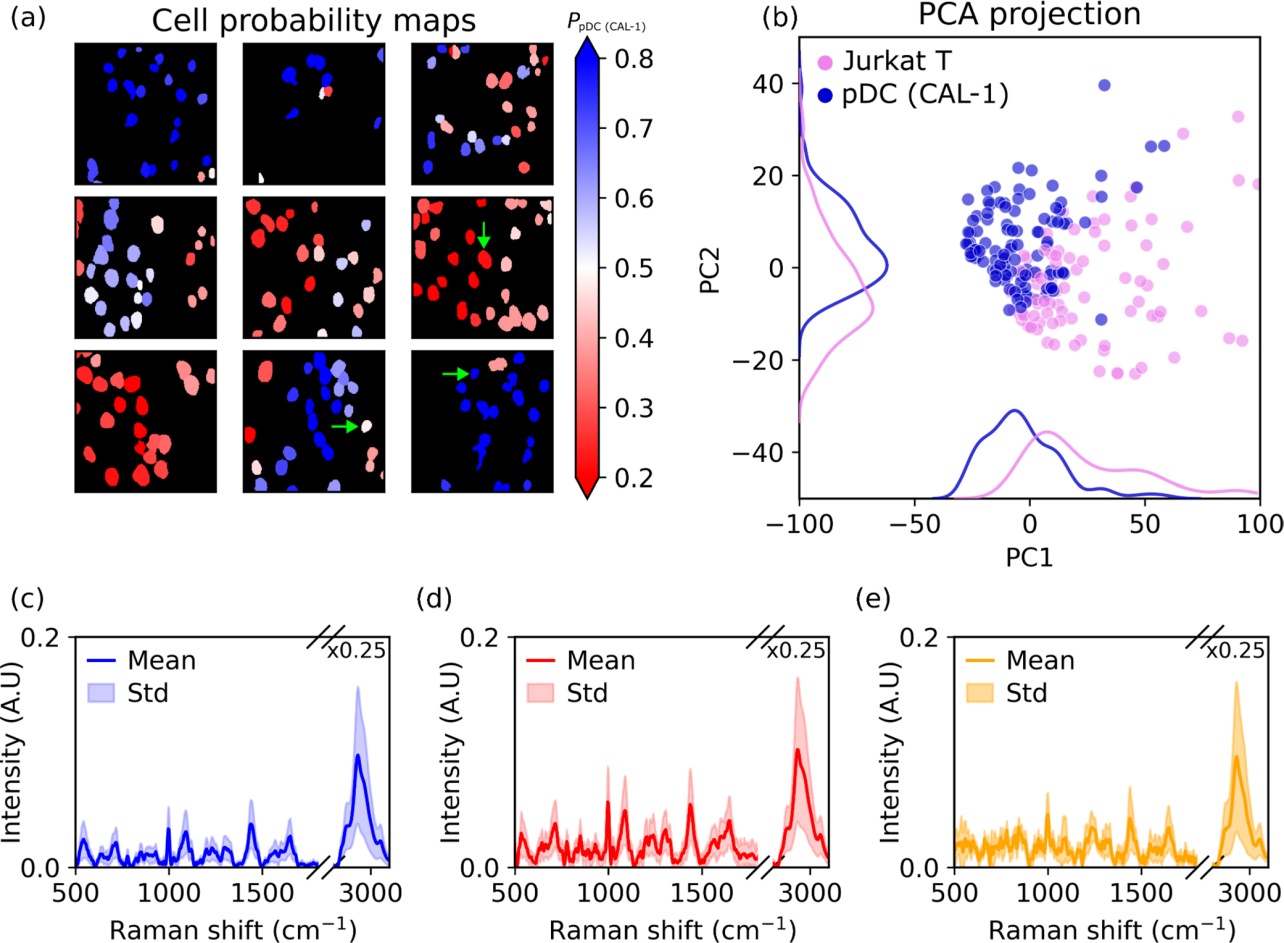
(a) Cell probability maps using the RF classifier trained using the labeled cell data, (b) PCA scores and kernel density estimates of each segmented cell on the principal component vectors obtained from the labeled data, (c) single‐cell Raman spectrum and variability of a pDC CAL‐1 high probability sample, (d) single‐cell Raman spectrum and variability of a Jurkat T high‐probability sample, (e) single‐cell Raman spectrum and variability of a low probability sample. Green arrows indicate cells shown in (c–e).

The result of the classification using the RF model on the unlabeled dataset is shown for nine different images in Figure 6a. The class probability for a single class (CAL‐1) denotes the color of each detected cell region. Cells with a probability of being a CAL‐1 > 0.5 were labeled as that type and vice versa. The magnitude of the class probability then essentially gives the likelihood, given the data and the model, that the class is indeed a CAL‐1 cell. We note there are some regions with a class probability around 0.5, and therefore, for that cell, the likelihood of classification to either class is low. We suspect that this results due to a low SNR for the cell, and we discuss this further below.

In Figure 6b, the projection of the classified mixed samples on the PCs from the labeled study is shown. The kernel density estimate of the distribution is also plotted. It can be seen that there is very similar separation of the data to the PCA score plot shown in Figure 4h. The relative location of the centroids of each cell type distribution is almost identical for both the labeled and unlabeled data, which signifies that the spectra share the same weighting among the first two PCs. This PCA cannot be taken as a validation of the classification of the unlabeled cells. However, it can be taken as an encouraging sign that the classified unlabeled spectra have similar characteristics to the labeled datasets. In order to analyze the results of classification, the expected proportion of cells classified was compared with the actual proportion measured within the mixture and using a Chi‐square test, a null hypothesis was formed. The null hypothesis was that there was no difference between the expected and observed cell frequencies. The *p*‐value of this test was 0.979, thus the null hypothesis was not rejected. Furthermore, the relative size distribution of the labeled and unlabeled data was available from the segmentation approach and was used to determine if the expected average size of cells was consistent with the known size (see Supporting Information S1).

In order to better elucidate why some cells are not classified with high probability, we investigate the individual “pixel” spectra within a given cell area for three specific cases: a cell with high prediction score for each cell line, and a third cell with a poor prediction score. The location of each cell is highlighted with a green arrow on Figure 6a. For all three cases, the mean and standard deviation spectra of the intra‐cell data are plotted. The results for the high‐probability pDC CAL‐1 and Jurkat T cells are shown in Figure 6c,d, and the low‐probability cell is shown in (Figure 6e). These spectra show that the intra‐cell variability is quite large, even for the high‐probability samples. This is possibly due to the natural macromolecular distribution of Raman scatterers within cells, which highlights one of the advantages of HSI classification, being the identification of the subcellular features that may contribute most to a difference between cell types. It can be seen in all cases that the CH‐stretching signals vary considerably. This is likely due to scattering effects due to the thickness variation across the cell. These data were not scatter corrected so as to visualize the variability in the cells. The training data however were scatter corrected. Of note is the difference between the mean spectrum of the low probability spectrum in (Figure 6e) and those of the two high probability spectra. In this spectrum, the fingerprint region was considerably lower in SNR, potentially due to an incorrect focal depth. There is also a larger variability of the spectrum in the region below 1000 cm^−1^ and in the PO_2_‐stretching band. An increased variability within a cell may indicate a single outlier pixel or more systematic differences in the noise levels within this cell.

### 6.4 Single‐Pixel Classification

**FIGURE 7:**
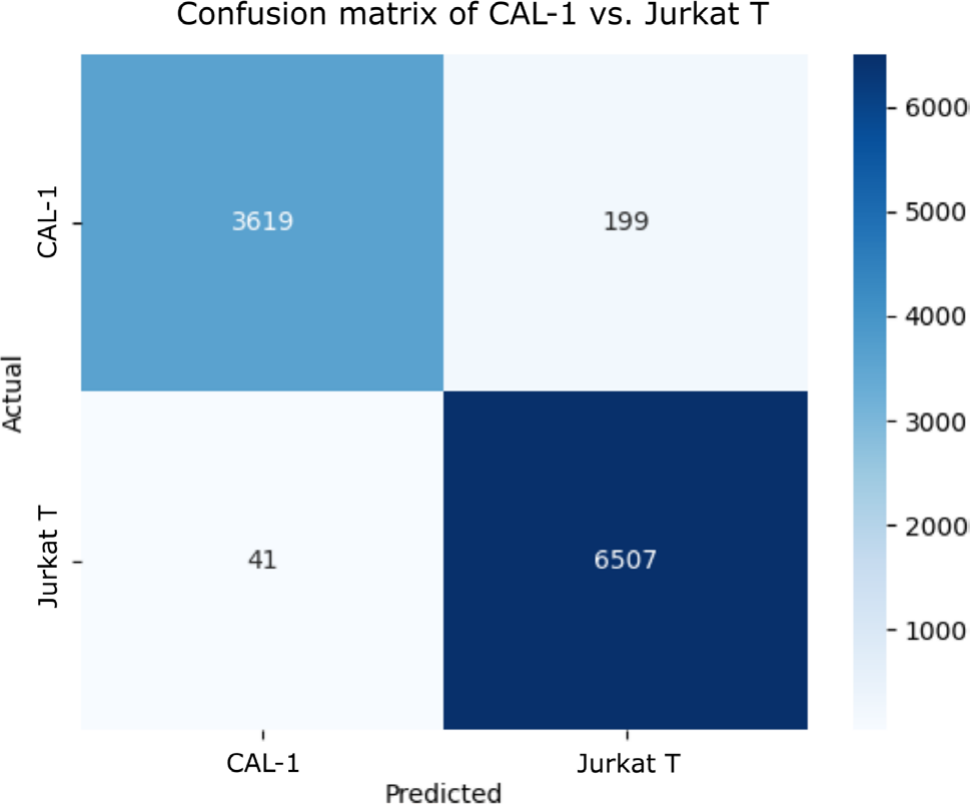
Confusion matrix for the classification of two cell lines using single‐pixel BCARS spectra, highlighting the classification accuracy.

To further advance our examination of BCARS immune cell classification, we shifted focus from averaged cell spectra to single‐pixel spectra with the aim of classification using shorter acquisitions. The traditional approach, while robust in validating our hypothesis regarding the effectiveness of using both fingerprint and CH‐stretch bands for BCARS classification, relies on aggregating spectra from all pixels within a cell's boundary. This method effectively results in an acquisition time similar to that of SR, typically around 1 s per cell, thus offering no obvious improvement in cell classification speed. To address this, we explored the potential of classifying cells based on single‐pixel spectra taken from the HSI, each with an acquisition time of 5 ms. This approach uses the same segmentation method as described previously, pooling spectra from the two distinct cell lines into respective training and validation sets. An RF model with the same parameters as used in the previous section was applied with a simplified single dataset partitioning for training and validation due to the computational demands associated with 10‐fold cross validation. A 90/10% train/test split was implemented to assess the classifier's performance. The results, as depicted in Figure 7, were quite promising, achieving a balanced accuracy of 97.1%. This compares favorably with the 99.4% balanced accuracy obtained from the whole‐cell data using 10‐fold cross‐validation, suggesting that rapid classification with single‐pixel spectra is possible, albeit with a small reduction in accuracy. Although 10‐fold cross‐validation was not feasible due to the extensive computational requirements, these findings indicate a significant step forward in speeding up the BCARS classification process.

## 7 Discussion

Our work demonstrates a fully automated high‐speed cell classification method for label‐free analysis. The retrieved Raman spectra obtained in this study show comparable signal content to SR spectra of leukocytes. Obtaining a high SNR BCARS cell spectrum for classification is nontrivial. Although a high signal intensity can be achieved via the nonlinear process, the SNR in the fingerprint region remains low and SVD is of paramount importance in pushing the signal‐to‐noise to a useful level. Combining both the SVD step and cell averaging step produced relatively high‐quality Raman spectra with easily identifiable vibrational features within the biologically relevant window from 600 to 3200 cm^−1^. It should be noted that when SVD is used to denoise a signal, there is an effective increasing of the exposure time in the experiment, since the information used to truncate the decomposed matrix is “learned” using the data itself. In essence, the power of SVD comes from the fact that there is a large distribution of signal with high similarity in the input data. Since such data are abundant in a HSI, there is no limitations to the use of SVD here; however, for classification of single cells where an HSI is not acquired, other denoising approaches would be necessary unless large datasets were recorded.

There was an apparent clustering of similarly labeled cells within the mixture sample as seen in Figure 6a. Although the data was pseudo‐labeled (according to prediction scores) and no ground truth is available, the clustering would not be expected in a mixture. The suspected reason for such result is due to a difference in the transport properties of each cell type which may have induced a spatial gradient of cell types from the center of the coverslip to the edge. This is supported by the fact that the average cell diameter was significantly different between the two types. Evidence to support this unexpected clustering of cell type on the mixed slide is provided in Supporting Information S1, where we use image segmentation to quantify cell size distribution of both the unlabeled and pseudo‐labeled data. There is clear evidence of grouping of cells according to cell size.

Reliably removing the NRB is critically important in any BCARS bio‐classification experiment. The laser characteristics such as its spectrum, power, and pulse duration are known to greatly affect the generation of the NRB, however, in a fiber laser, the stability of the sources can be remarkably high. In order to prevent any potential confounding by the laser system, all experiments in this study were performed on the same day and with as little time as possible between acquisitions. Nevertheless, the NRB will change from one point to the next. In the context of full automation, the phase retrieval step was the only supervised stage of the preprocessing, since the phase detrending parameters must match the NRB strength in order to prevent over/underfitting to the resonant susceptibility phase. It was observed that once optimization was performed on a single representative spectrum (for either cell class), the KK procedure produced consistent results over the whole experiment. It is important in a BCARS bio‐classification study to have confidence that spectral classification results from resonant differences in the spectra and not from variation introduced by NRB. Differences in the spectral datasets, as quantified by PCA, appear to occur at characteristic Raman shifts corresponding to prevalent biomolecules, which is typically used to confirm that chemical features discriminate the cells.

The results demonstrate that BCARS can be used to obtain high‐content chemical information from cells on the order of 5 ms per pixel. At the image resolution used (1 μm), this resulted in a scan time of approximately 300 s (there was 0.5 s of additional dwell time between each fast‐axis scan in order to read data). We selected our step size to balance scan time with image quality, allowing for sufficient spectral measurements per cell to minimize sampling errors and improve classifier accuracy. Theoretically, the finest spatial resolution adheres to the diffraction limit, calculated as $\lambda /\left(2\mathrm{NA}\right)\approx 321$ nm. While achievable, using this finer step size would likely offer minimal gains in classification accuracy. This scan rate enables 1 mm^2^ to be scanned in 1.38 h provided the data could be transferred after the scan. The main advantage of this method over SR is obviously the ability to record HSIs of microscopic areas with much greater speed. We are confident that the pixel exposure time could be reduced to around 1 ms, with increases in the number of spectra recorded, for use in the SVD preprocessing step. In addition to producing a better average spectrum compared with SR, which typically applies point scanning, another benefit over SR is the lack of any fluorescence and the ability to use low NA collection optics. The latter is especially attractive for imaging glass slides or through flow apparatus.

The approach we have taken here to classify cells can also be contrasted to the use of SRS imaging to obtain similar information. SRS imaging studies have been limited to narrow bandwidths such as the high‐wavenumber region, since broadband detection is not practical. Using this information however, label‐free cell imaging is possible [43]. While the chemical information may be reduced compared with full fingerprint analyses, the lack of any interfering NRB in SRS is one reason why it affords relatively little processing in order to achieve subcellular imaging. Obtaining the full biological frequency band for a cell using SRS would require wavelength tuning over 3500 cm^−1^, which can be timely. The lack of NRB affords SRS an advantage over BCARS in terms of quantitative accuracy; however, instantaneous SRS methods require complex systems with parallelized lock in amplifiers and are typically limited to recording the upper wavenumbers alone. It has not been demonstrated that this band can provide for cellular classification studies in the same way as the Raman fingerprint region.

Fundamental to this study was the level of confidence observed in the classification of the unlabeled data. Although supervised testing showed very high balanced accuracy on a test set, there was no definitive way to quantify accuracy on the unlabeled data. Instead, two semiquantitative methods were used to infer the likelihood of a successful implementation on the unlabeled data; PCA and size analysis. The projection of the unlabeled data on to the PCs obtained from the labeled data showed very good agreement between cell types. This is indicative that the pseudo‐labeled data varied in similar way to the known labels. In order to gain a deeper understanding of the result, a sizing analysis was also performed and it was shown that the cell diameter was statistically different for the two labeled cell types. The average cell diameter was then compared between the labeled and unlabeled data and was not significantly different for the Jurkat T cells; however, there was a significant difference between the pDC CAL‐1 cells across experiments. We suspect that this is due to some misclassifications of this type. There was however the same signed significant difference of cell diameters for both the labeled data and unlabeled data which is more evidence toward a successful classification.

A key feature of this work is the spectral averaging of the whole BCARS spectrum over each cell. Since the chemical information is expected to be much more informative than the morphological information, in a general sense, the chemical averaging was deemed appropriate. However, since morphological information is always present in the HSI, it could also be used in improving the classification accuracy. Some features may simply be calculated based on the image and appended to the spectrum as added variables, for example, cell diameter, ellipticity, or convexity. If a higher resolution was used, a deeper model could be developed that uses the full‐cell hypercube to classify the whole cell rather than a single spectrum; however, this would require a bespoke DL architecture. Obtaining the single‐cell spectrum was done using the mean over the whole cell extent. This was considered the most common method of obtaining a representative spectrum from the cell; however, there are several potential improvements that could be studied in future iterations of this work. One example is to remove outliers prior to calculating the average, using some statistical metric. This would prevent single pixels that are not representative of the cell from perturbing the result. Outliers of this type may be due to abnormal cell morphology such as complex shapes or could be due to errors in the segmentation or NRB removal step. There is also the potential to use the median Raman spectrum as the representative spectrum, which should reduce the effect of outliers.

The single‐cell classification method described here is not without its drawbacks; however, and there are still improvements that can be made experimentally. First, the NRB can require manual intervention when the resonant to nonresonant susceptibility ratio changes as discussed earlier. This issue may be mitigated through the use of DL methods. Second, some cells can prove to be problematic, likely due to a thin morphology that is partially missed by the highly focused laser pulses resulting in low SNR even with SVD denoising and cell averaging. One potential improvement in this regard, might be to apply a dynamic focusing methodology making use of real‐time deformable mirrors.

In terms of future work, the exploration of high‐speed single BCARS spectra for rapid cell classification highlights the potential of this technology for high‐throughput cellular analysis. By reducing the acquisition time to just 5 ms per spectrum, we have significantly reduced the time usually associated with Raman cytology. This advancement could be particularly important for flow cytometry applications, where the ability to quickly process large volumes of cells is necessary. Furthermore, the application of this technique to broad or wide grid hyperspectral imaging could also enable tissue diagnostics in fast scan times. This method could also augment the growing field of SRS histology for virtual staining [44]. The current approach is to use just two wavenumbers relating to CH_2_ and CH_3_. This two‐wavenumber HSI can be used to generate H&E‐like virtually stained images that can then be interpreted by a pathologist during surgery or by machine learning. Although extremely powerful, this approach is still based on spatial information in the sense of morphological distributions. It is not based on chemical classification, which is the basis of the approach used here. On the basis that 5 ms acquisitions can potentially be used for cell classification, we envisage that classification of diseased tissue based on biochemical difference could be achieved using broad or wide grid hyperspectral imaging in similar scan times required in SRS histology.

## 8 Conclusions

This work demonstrates, to the best of our knowledge, the first experimental classification of a mixture of two human‐derived cell lines, a pDC (CAL‐1) and a T‐cell (Jurkat) line, using BCARS hyperspectral imaging. The average Raman spectrum of each cell was then used to classify different cell types. The acquisition procedure was fully automated, whereby optimal parameters required in preprocessing such as for the NRB removal approach (KK/DL, etc.) could be determined for some initial data but do not require any post hoc tuning. Furthermore, we do not expect these parameters to vary between cell types and days. The optimization of such hyperparameters; however, may decrease batch effects such as systematic bias from NRB removal and changes in the laser spectrum, which could potentially increase the diagnostic accuracy.

Our approach is well‐suited to high‐throughput cell analysis where cells are deposited on a microscope slide, such as for routine clinical cytopathology. Additional testing on a larger number of cells, using a second in situ analytic technique such as a histochemical/cytochemical stain or fluorescence imaging could be performed to further validate the results, although these analytical methods are usually nonspecific to cell type. Fluorescence in situ hybridization (FISH) could be used to improve specificity using a gene‐specific marker, at the expense of confounding by the labeling procedure, which must be done separately prior to mixing and thus may alter the BCARS signal. The main advantage of our approach over a traditional cell targeting approach using spectroscopy is the amount of data available for analysis and there being no need for a separate widefield acquisition to enable the targeting. The morphological information present in the HSI can also be used to further understand the sample, and coupled with chemical contrast, can provide complimentary information that can aid in the evaluation of a classifier.

## Conflicts of Interest

The authors declare no conflicts of interest.

## Supporting information


**Data S1** Supporting Information.

## Data Availability

The data that support the findings of this study are available from the corresponding author upon reasonable request.
