# Supplementary material for: Broadband CARS Hyperspectral Classification of Single Immune Cells
Source: J Biophotonics. 2025 Jan 18;18(3):e202400382. doi: 10.1002/jbio.202400382 (PMC11884967; doi:10.1002/jbio.202400382)
Supplement: Supplementary file 1 — Data S1 Supporting Information. [file JBIO-18-e202400382-s001.pdf]

Broadband CARS hyperspectral classification of single immune cells: supplemental document

1. RANDOM FOREST CLASSIFIER HYPERPARAMETER OPTIMIZATION

Hyperparameter optimization was performed for the supervised model using a grid search. The only parameter optimized was the number of trees in the model. This parameter determines the complexity of the model, however for best generalization performance, its value was kept as small as possible while maintaining good accuracy. The number of trees was varied from 1 to 200 and the mean balanced accuracy was calculated using 10-fold cross validation. A value of 40 trees was chosen since the balanced accuracy appeared to be relatively constant after this value. The results are shown below.

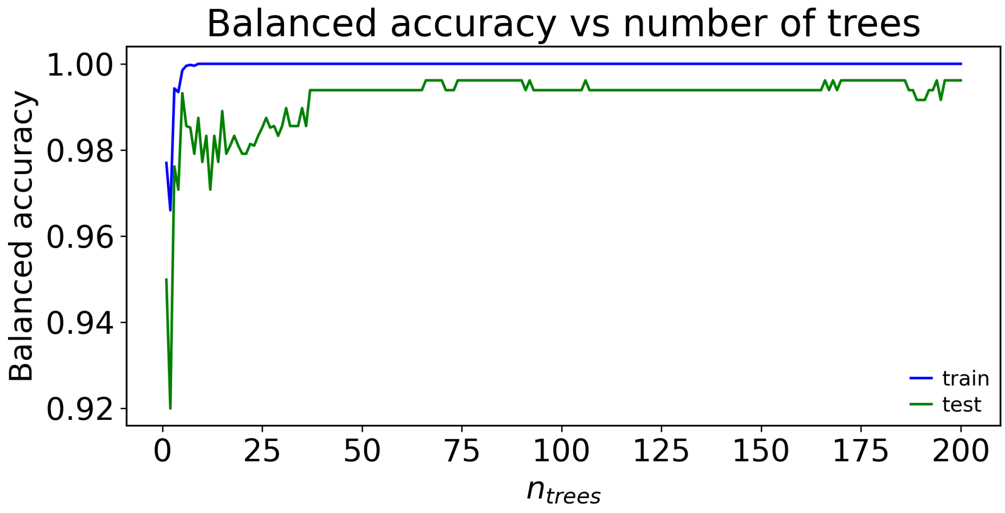

Fig. S1. Results of cross-validation to determine the optimum number of trees in the Random Forest model.

2. BRIGHTFIELD IMAGING OF INDEPENDENT AND MIXED SAMPLES

Brightfield refection images of the independent cell types are shown in Fig. S2. Also shown is an image of the mixture. As explained in the main text, there was an apparent clustering of cell types based on size (and thus species, see section 3), most likely due to diffusion effects during the drying of the solution on to the coverslip. Since a dry-mount was used, the samples may have separated on the coverslip due to their different mobility (size). It was not possible to mitigate this effect in this experiment due to the mounting approach used, however we expect it should not affect the results.

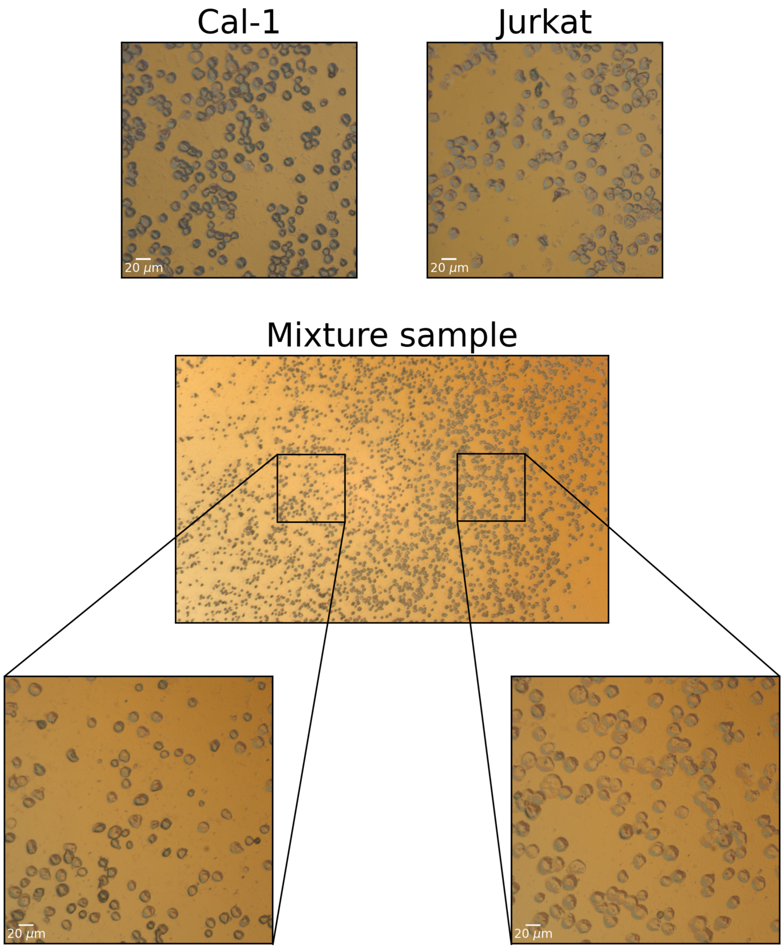

Fig. S2. Brightfield images of the independent cell types and the mixture.

3. SIZE ANALYSIS OF CELLS USING IMAGE-BASED SEGMENTATION

The cell segmentation step produced area masks from which the cell sizes could be deduced. The distribution of the diameters for each cell type and both experiments (labelled and unlabelled) is shown in Fig. S3 for cells that had a prediction probability of being a CAL-1 of $0.7 < p < 0.3$. Thus, only cells with a high confidence of classification were used in the size analysis. It can be seen that the labelled data had a significant difference in the cell diameter between cell types. Jurkat T cells had a median diameter of 17.3 μm, while CAL-1 cells had a median diameter of 11.9 μm. Across experiments, the cell diameter was not statistically different between the Jurkat T cells, however it was for the CAL-1 cells. This may be due to some actual CAL-1 cells being classified as Jurkat T cells.

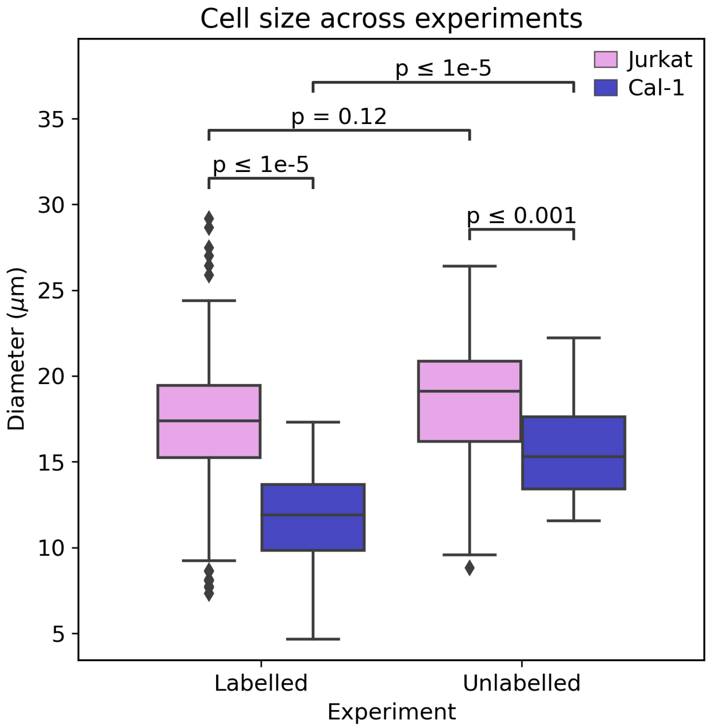

Fig. S3. Boxplot of the cell diameter across species and experiments. An independent sample t-test was used to determine if the difference between mean diameters was statistically significant.
